# Changes in HIV prevention programme outcomes among key populations in Kenya: Data from periodic surveys

**DOI:** 10.1371/journal.pone.0203784

**Published:** 2018-09-19

**Authors:** Helgar Musyoki, Parinita Bhattacharjee, Andrea K. Blanchard, Japheth Kioko, Shem Kaosa, John Anthony, Prakash Javalkar, Janet Musimbi, Serah Joy Malaba, Carol Olwande, James F. Blanchard, Martin Sirengo, Shajy Isac, Stephen Moses

**Affiliations:** 1 National AIDS and STI Control Programme, Ministry of Health, Government of Kenya, Nairobi, Kenya; 2 Centre for Global Public Health, Department of Community Health Sciences, University of Manitoba, Winnipeg, Canada; 3 Partners for Health and Development in Africa, Nairobi, Kenya; 4 Karnataka Health Promotion Trust, Bangalore, India; International AIDS Vaccine Initiative, UNITED STATES

## Abstract

The Kenya National AIDS and STI Control Programme (NASCOP) conducted annual polling booth surveys (PBS) in 2014 and 2015 to measure outcomes from the national HIV prevention programme for key populations (KPs), comprising behavioural, biomedical and structural interventions. KPs included female sex workers (FSWs), men who have sex with men (MSM) and people who inject drugs (PWID). We compared survey results from the first and second rounds. Comparing the second to the first round, significantly more FSWs (93% vs. 88%, p<0.001) and MSM (77% vs. 58%, p<0.001) reported condom use at last sex with a paying client, and at last anal sex among MSM (80% vs. 77%, p<0.05) and PWID (48% vs. 27%, p<0.01). However, condom use with regular partners remained low, at less than 53% for FSWs and 69% for MSM. Among PWID, there was a significant increase in use of new needles and syringes at last injection (93% vs. 88%, p<0.001), and a significant decrease in reported non-availability of clean needles (23% vs. 36%, p<0.001). The number of overdoses in the past six months reduced significantly but remained high (40% vs. 51%, p<0.001). FSWs and MSM reported significantly higher HIV testing, and in all KP groups, over 93% reported ever having been tested for HIV. Among the respondents self-reporting to have tested HIV positive (24% of FSW, 22% of MSM and 19% of PWID), 80% of FSWs, 70% of MSM, and 73% of PWID reported currently taking antiretroviral therapy (ART). While the experience of forced intercourse by partners declined among FSWs (18% vs. 22%, p<0.01) and MSM (13% vs. 17%, p<0.01), more FSWs reported violence by law enforcement personnel (49% vs. 44%, p<0.001). These findings provide valuable information on the programme’s progress, and a signpost for the integrated behavioural, biomedical and structural interventions to achieve their HIV prevention targets.

## Introduction

The heterogeneity of the HIV epidemic globally necessitates an approach that allows geographic and population-based prioritization to achieve efficient planning and implementation of the response [1]. However, the adoption of such an approach remains uneven, as many of those who are most at risk for HIV infection continue to be left behind [2]. Recent studies suggest that the likelihood of acquiring HIV is 24 times higher among people who inject drugs (PWID) than adults in the general population, 10 times higher among female sex workers (FSWs), and 24 times higher among men who have sex with men (MSM) [2]. UNAIDS recommends that to reduce new HIV infections to an annual global target of 500,000 by 2020, and achieve the ambitious 90-90-90 treatment targets [3], an intensive focus on combination prevention approaches is required. This involves evidence-informed and human rights-based HIV prevention programmes, with a combination of biomedical services, and behavioural and structural interventions, aiming for community empowerment among key populations [2].

In response to the HIV epidemic in Kenya, the Kenya AIDS Strategic Framework 2014/15–2018/19 (KASF) prioritizes the key populations of FSWs, MSM and PWID [4, 5]. As described in detail in a previous publication, HIV prevalence among these key populations (KP) is high [5]. A survey conducted in 2010 showed that 29% of FSWs, 18% of MSM and 19% of PWID were HIV positive [5, 6]. The National AIDS Control Council (NACC) modes of transmission study conducted in 2009 showed that 33% of all new HIV infections in Kenya occurred among key populations [7]. The Kenyan epidemic is socially and geographically heterogeneous as well, with certain counties and populations having higher HIV prevalence and incidence than others. Hence, selected counties and populations are being prioritized for scaled-up intensive HIV prevention programmes [4]. Kenya conducted a national mapping exercise of key populations in 2012, and through a consensus meeting, estimated that there were approximately 133,675 FSWs, 18,460 MSM and 18,327 PWID [8]. This exercise also provided county and “hotspot”-wise estimations, providing a road map for Kenya to scale up its key population programmes by prioritizing those counties with higher numbers of people in key population groups. In recent years, the U.S. President’s Emergency Fund for AIDS Relief (PEPFAR) and the Global Fund against AIDS, TB and Malaria (GFATM) have expanded their funding to 82 implementing partners, covering 123,113 FSWs, 24,958 MSM and 13,315 PWID across 32 of Kenya’s 47 counties [9].

Kenya’s national HIV programme has developed several policies, guidelines and training curricula to standardize programme scale-up for KPs. Kenya has also sought to create an enabling environment through structural interventions to widen access to health services for KPs and heighten community mobilization, particularly to address a context in Kenya where sex work, same sex relationships and drug use is criminalized. The KP services therefore comprise a combination prevention approach [4] that includes not only biomedical and behavioural, but also structural interventions, following global guidance [10] for programming for key populations [11, 5]. This approach is necessary to achieve the targets set by the country to reduce new infections, AIDS-related mortality and HIV-related stigma and discrimination by 2018/19 [4].

The KASF’s monitoring and evaluation framework includes annual behavioural surveys among key populations in Kenya to monitor programme outcomes. Specifically, Kenya uses a polling booth survey (PBS) methodology to generate relevant indicators, through an anonymous group interview process [5]. The first PBS was conducted in 2013–14 as a baseline for monitoring HIV-related risk behaviour among FSWs, MSM and PWID, their utilization of existing prevention and care programmes, and structural issues related to KPs’ vulnerability to HIV. The findings of the first survey have been previously published, and they describe KP’s HIV risk and prevention behaviours, utilization of existing programmes and services, and experiences of violence [5]. The second round of the PBS was completed in 2015. The objective of this paper is to compare the results of the first and second polling booth survey rounds, to identify where progress has been made, and where further attention is required by the HIV/AIDS key populations programmes in Kenya. Studying outcomes nationally across different key population groups will also make a contribution to global research evaluating key population HIV/AIDS interventions, which has predominantly focused on a single location or population to date.

## Methodology

### Study design and sample selection

The study methodology for the survey in 2015 was similar to 2013–14, using the PBS methodology previously described [5]. However, the geographic coverage for FSW and PWID surveys was expanded in the follow-up round. In addition to the seven study locations included in the 2013–14 baseline survey (Nairobi, Mombasa, Nakuru, Nyeri, Kiambu, Kisumu and Eldoret) for FSWs, four other geographic areas were added (Homabay, Bungoma, Meru and Kitui). For PWID, in addition to the two study locations in the 2013–14 baseline survey (Mombasa and Nairobi), two other areas (Kilifi and Kisumu) were added. For MSM, the five locations (Nairobi, Mombasa, Kisumu, Nakuru and Kiambu) were the same in the 2013–14 baseline survey and the follow-up round in 2015. The expansion of FSW and PWID geographic coverage was done to account for regional heterogeneity in terms of typology of KPs, HIV prevalence and socio-cultural contexts.

As previously described [5], a two-stage, stratified cluster sampling methodology was used to recruit PBS participants. For the first stage of sampling in each study location, “hotspots” were selected as the primary sampling units (PSUs) from the list of all active and mapped hotspots, which served as the sampling frame. A hotspot was defined as any physical location where KP members meet partners or clients (soliciting or cruising), engage in sexual intercourse with partners/clients, or obtain or inject drugs. The lists of hotspots were then stratified by geographic location and typology (i.e. FSW, MSM or PWID), before selecting the PSUs systematically. It is important to note that hotspots where KP estimates were small were merged with nearby hotspots of the same typology, so that an adequate number of KPs would be available within each PSU. The number of PSUs per study site was determined based upon the required sample size and the average number of KPs per PBS session.

Sample sizes were calculated to detect a 15% change in the indicators of interest between the baseline and follow-up surveys (e.g. a 15% increase in condom use with casual clients from a baseline of 50%), with 80% statistical power. The target sample size for each KP group across all study sites (accounting for a 15% non-response rate) was 3,845 FSWs, 1,528 MSM and 1,087 PWID. In the second stage of sampling, peer educators who had received thorough training on the sampling procedure were asked to develop a list of individuals self-identifying as key population members among those who frequented the identified sampling hotspots [8]. To reduce selection and participation biases, peer educators used a systematic random sampling method [12, 13] to identify potential participants that frequented hotspots, rather than focusing only on individuals who regularly accessed services provided by intervention programmes. Potential participants were made aware that refusal to participate would not, under any circumstances, jeopardize their ability to utilize services provided by programmes. Individuals who agreed to participate were provided with information on the date, time, and venue of their PBS session, which was arranged in consultation with implementing non-governmental or community-based organizations in each study site. Up to twelve participants were organized into homogenous groups based on socio-demographic and geographic characteristics. A total of 457 PBS group sessions were conducted. All PBS sessions were led by a field researcher, as well as research assistants who self-identified as KP members.

### Data collection and analysis

PBS is a group interview method in which participants are provided a private space or booth containing colour-coded ‘yes’, ‘no’, and ‘not applicable’ ballot boxes, and a set of numbered ‘voting’ tokens corresponding to each questionnaire item. Participants then answer survey questions that are read aloud by the researcher by placing the appropriately numbered token in the relevant ballot box, as described in more detail elsewhere [5]. There is evidence that Polling Booth Surveys provide more accurate reporting of sensitive sexual behaviours due to the anonymity provided to the respondents, whereas both face-to-face interviews and self-administered questionnaires can be prone to more social desirability, or non-disclosure bias, due to perceived lack of confidentiality [14, 15]. Additionally, PBS is a simple method to implement, and allows participation from key populations in designing and administering the questions. In the current survey, key population members were involved as research assistants and researchers to administer the survey, along with external researchers.

Three different questionnaires were used in the PBS sessions for the FSW, MSM, and PWID respondents, respectively. As a first follow-up to the baseline survey, the second-round questionnaires retained the questions used at baseline, allowing for the measurement of changes in outcomes related to behavioural, biomedical, and structural interventions over time. New questions added in the second round included HIV status, screening and uptake of hepatitis vaccine, and exposure to peer education. The final survey tools were translated from English to Kiswahili, Kenya’s official language, then back-translated to English to ensure accuracy and consistency. The tools were tested with key populations, and adjustments were made based on their feedback prior to the final data collection.

After data collection, data were entered using Microsoft Access and exported to SPSS (v20) and Microsoft Excel for analysis. Descriptive analysis was performed to produce frequencies, proportions and comparative statistics. Given that PBS data are anonymized and aggregated at the group level (i.e. responses are summarised for each PBS session), the data are unlinked, and do not allow for more complex, multivariate analyses. Data were weighted using overall and hotspot level selection probabilities, taking into account the sample size and KP population size estimates, to provide estimates at the overall level. Overall selection probability was derived using the total sample size and the estimated size of KPs across the consistent locations, whereas the selection probability at the hotspot level was derived using the sample size and the estimated size of the KP population at the respective hotspot. The final weights were calculated using both overall sampling probability and the selection probability in each hotspot. The comparative findings presented in this paper compare the data from only the consistent geographic locations in the baseline and first follow-up survey. Chi square tests of significance were conducted to assess changes over time. There were seven consistent locations for FSWs (Nairobi, Mombasa, Kisumu, Nakuru, Kiambu, Eldoret and Nyeri), five for MSM (Nairobi, Mombasa, Kisumu, Nakuru and Kiambu) and two for PWID (Nairobi and Mombasa). The complete findings for all locations are available separately [16]. The data for the 2014 PBS survey were presented in our previous publication [5]. Some data from the 2014 survey have also been presented in this paper to compare them with the results of the 2015 survey.

### Ethical considerations

Kenya’s Ministry of Health has approved the PBS as a method for routinely collecting data to inform programme monitoring and evaluation. Participation in PBS sessions was voluntary. Women and men, at least 18 years of age, and who identified as KPs, were eligible to participate in the survey, after providing written informed consent similar to the practice followed in 2014. Data were anonymous and unlinked, thus minimizing potential risks associated with participation such as disclosure of HIV status. No incentives were provided to participants for taking part in the surveys, although costs of travelling to and from the PBS session sites were reimbursed. The de-identified data collected for this study was part of routine monitoring by the Government of Kenya of their HIV programme and is publically available on a NASCOP electronic database. Ethical approval to conduct secondary analysis of the PBS data was received from Kenyatta National Hospital–University of Nairobi Ethical Review Committee, approval number P647/11/2017.

## Results

### Socio-demographic characteristics and reported risk behaviours

The overall response rate was 95% in 2015 and 99% in 2014. As shown in Table 1, a total of 5,446 and 4,080 participants took part in round 1 and round 2 of the PBS respectively. The sample size in round 1 was higher for FSWs, as we used lower baseline values for condom use for the two locations (Nairobi and Mombasa) in calculating the required sample size.

**Table 1:** Polling booth survey participants in consistent study locations, 2014 and 2015 by KP category.

	Number of Participants—PBS 2014				Number of Participants—PBS 2015			
	FSWs	MSM	PWID	Total	FSWs	MSM	PWID	Total
**Consistent locations**	3,448	1,308	690	5,446	2,228	1,254	598	4,080

As shown in Table 2 below, the majority of the FSW respondents in the 2015 survey were in the age group 26–40 years (64%), while the majority of the MSM respondents were in the age group 18–25 years (67%). For PWID, the majority were in the age group 26–40 years (70%). The age distribution of FSW respondents changed slightly from the first survey, with the proportion of FSW respondents in the age group 26–40 years increasing from 53% in 2014 to 64% in 2015. The age distributions of the MSM and PWID did not change.

**Table 2:** Socio-demographic characteristics of polling booth survey participants in consistent study locations, 2014 and 2015.

	PBS 2014			PBS 2015		
	FSWs n = 3,448 (%)	MSM n = 1,308 (%)	PWID n = 690 (%)	FSWs n = 2,228 (%)	MSM n = 1,254 (%)	PWID, n = 598 (%)
**PBS sessions total**	204	70	63	272	108	77
**Age range (years)**						
18–25	1,495 (43%)	855 (65%)	112 (16%)	699 (31%)	843 (67%)	97 (16%)
26–40	1,839 (53%)	425 (32%)	485 (70%)	1,437 (64%)	408 (32%)	420 (70%)
Above 40	114 (3%)	28 (2%)	92 (13%)	92 (4%)	3 (.2%)	81 (13%)
**Sex**						
Male	**-**	1,308 (100%)	608 (88%)	**-**	1,254 (100%)	468 (78%)
Female	3,448 (100%)	-	82 (11%)	2,228 (100%)	**-**	130 (22%)

Table 3 shows the percentages of self-reported risk behaviours in 2015. Among MSM, 69% had ever exchanged sex with other men for money or other goods, and 60% had exchanged sex for goods or money in the last month. 35% of PWID who were females had sex with paying clients in the last 3 months, and 24% of men who injected drugs paid for sex in the past one month. 29% of MSM also had a non-paying female partner. 59% of FSWs and 68% of MSM had regular non-paying male partners. 63% of men who injected drugs had regular non-paying female partners, and 70% of females who injected drugs had regular male non-paying partners. 12% of FSW respondents, 87% of MSM respondents and 5% of PWID respondents reported having anal sex in the past month. 94% of men and 82% of women who inject drugs reported using heroin or narcotic drugs in the past month, while 6% of FSWs and 10% of MSM reported injecting heroin or narcotic drugs in their lifetime.

**Table 3:** Self-reported risk behaviours of polling booth survey 2015 participants in consistent locations.

Risk behaviour	Participants from consistent locations, 2015			
	FSWs, n = 2,228 (%)	MSM, n = 1,254 (%)	PWID, n = 598 (%)	
			Male	Female
**Exchanged sex for money/goods**				
Ever exchanged sex with other men for goods or money	2,228 (100%)	857 (69%)	-	-
Exchanged sex with other men for goods or money in last one month	-	680 (60%)	-	-
Exchanged sex with any paying client in the past three months	-	-	19 (8%)	36 (35%)
Paid for sex in past one month	-	-	111 (24%)	9 (7%)
**Regular non-paying partner**				
Have a female regular non-paying partner	-	349 (29%)	294 (63%)	-
Have a male regular non-paying partner	1,322 (59%)	845 (68%)	-	91 (70%)
**Anal sex**				
Had anal sex in the past one month	256 (12%)	1,097 (87%)	21 (5%)	5 (5%)
**Heroin/ narcotic drug use**				
Ever injected heroin or narcotic drugs	139 (6%)	122 (10%)	-	-
Injected heroin or narcotic drugs in the past month	-	-	441 (94%)	107 (82%)

### Behavioural outcomes

#### Condom use

As shown in Table 4, comparing the second to the first round of the PBS in the consistent locations, a significantly greater proportion of FSWs (93% vs. 88%, p<0.001) in the second round reported using a condom at last sex with a paying client. Similarly, there was an increase in condom use at last sex with a paying client among MSM (77% vs. 58%, p<0.001). There was no change among PWID related to condom use with a paying client (68% vs. 67%, p = 0.9). Condom use at last sex with regular, non-paying partners continued to be lower than with paying partners across all KP groups. However, in the second round of the PBS, condom use at last sex with regular non-paying male partners declined slightly (53% vs 57%, p<0.01) among FSWs. There was no change in reported condom use among MSM (69% vs. 70%, p = 0.8) with male regular partners. There was also no significant change in condom use at last sex with a regular non-paying female partner for MSM respondents (68% vs. 70%, p = 0.5). Overall, there was a significantly higher proportion of MSM (80% vs. 77%, p<0.03) and PWID (48% vs. 27%, p<0.01) reporting condom use at last anal sex act with partners in the second round. Condom breakage and slippage reduced significantly among FSWs (24% vs. 28%, p<0.002) and MSM (16% vs. 20%, p<0.01) in the consistent locations, but not significantly among PWID (20% vs. 23%, p = 0.2).

**Table 4:** Self-reported preventive behaviours among polling booth survey participants, 2014 and 2015 in consistent locations.

Behaviours	FSWs			MSM			PWID				
	Consistent locations			Consistent locations			Consistent locations			2015	
	2014 (%)	2015 (%)	p-value	2014 (%)	2015 (%)	p-value	2014 (%)	2015 (%)	p-value	Male (%)	Female (%)
**Condom use**											
Condom use at last sex with any paying client	88	93	p<0.001	58	77	p<0.001	67	68	p = 0.9	57	84
Condom use at last sex with a regular, non-paying male partner	57	53	p<0.01	70	69	p = 0.8	-	-	-	-	-
Condom use at last sex with a regular, non-paying female partner	-	-	-	70	68	p = 0.5	-	-		-	-
Condom use at last anal sex	58	55	p = 0.3	77	80	P<0.05	27	48	p<0.01	41	61
Condom breakage and slippage	28	24	p<0.001	20	16	p<0.01	23	20	p = 0.2	19	24
**Condom non-use**											
At least one occasion of unprotected sex with a paying client in the past month	36	25	p<0.001	-	-	-	-	-	-	-	-
Partner did not want to wear condom in the past month	31	27	p<0.001	23	23	p = 0.7	-	-	-	-	-
Condom not available at time of sex in the past month	23	17	p<0.001	33	25	p<0.001	35	32	p = 0.3	34	25
One or both partners consumed alcohol	26	20	p<0.01	30	26	p<0.05	-	-	-	-	-
Client offered more money for condom non-use	21	21	p = 0.9	-	19	-	-	-	-	-	-
**Needle and syringe usage**											
Used new needle and syringe when injecting drugs last time	-	-	-	-	-	-	88	93	p<0.001	93	91
Shared needle at last injection	-	-	-	-	-	-	17	10	P<0.001	8	18
Clean needle not available in the past month	-	-	-	-	-	-	36	23	p<0.001	22	23
**Drug overdose**											
Overdosed in the last 6 months	-	-	-	-	-	-	51	40	p<0.001	43	33

#### Condom non-use

Condom non-use among FSWs reduced significantly in the consistent sites. Only a quarter of FSWs reported having at least one occasion of unprotected sex with a paying client in the past month in the second round (25% vs. 36%, p<0.001). There was also a significant decrease in condom non-use among FSWs (27% vs. 31%, p<0.001), due to the partner not agreeing to wear a condom. There was a significant reduction in reports of condom non-availability at the time of sex in the past month among FSWs (23% vs. 27%, p<0.001) and MSM (25% vs. 33%, p<0.001). Alcohol consumption during sex also reduced significantly among FSWs (20% vs. 26%, p<0.001) and MSM (26% vs. 30%, p<0.05).

#### Needle and syringe usage, including overdose

More than 90% of PWID reported using a clean needle and syringe during their most recent injection. This is a significant increase in the consistent locations from the first round of PBS (93% vs. 88%, p<0.002). Only 10% of the PWID reported sharing their last injection, a significant reduction from the previous round (10% vs. 17%, p<0.001). There was also a reduction in PWID reporting that there had been at least one occasion in the past one month when clean needles were not available at the time of injection (23% vs. 36%, p<0.001). Although there was a reduction in reported drug overdose among PWID (40% vs. 51%, p<0.001), reported overdose remained quite high.

#### Exposure to peer education

Peer education is an important component of behavioural interventions, so exposure to peer education was added in the second survey as an outcome measure. As shown in Table 5, 79% of FSWs, 73% of MSM and 85% of PWID reported that they had been met by peer educators in the last three months.

**Table 5:** Self-reported programme exposure and awareness among polling booth survey participants, 2014 and 2015 in consistent locations.

Behaviours	FSWs			MSM			PWID				
	Consistent locations			Consistent locations			Consistent locations			2015	
	2014 (%)	2015 (%)	p-value	2014 (%)	2015 (%)	p-value	2014 (%)	2015 (%)	p-value	Male (%)	Female (%)
**Exposure to peer education**											
Met by a peer educator in last 3 months	-	79	-	-	73	-	-	85	-	81	90
**Knowledge**											
Know that HIV can be transmitted through anal sex	81	86	p<0.001	90	91	p = 0.4	-	80	-	86	58
Condoms can protect against HIV transmission	81	90	p<0.001	95	90	p<0.001	86	76	p<0.001	75	79

#### Knowledge about HIV

The majority of PBS participants knew that HIV is transmitted through anal sex, with a significant increase among FSW respondents between the two rounds (86% vs. 81%, p<0.001). While there was an increase in reported knowledge about condoms protecting against HIV transmission among FSWs (90% vs. 81%, p<0.001), there was a decrease in reported knowledge about condoms protecting against HIV transmission through anal sex among MSM (90% vs. 95%, p<0.001) and PWID respondents (76% vs. 86%, p<0.001).

### Health service outcomes

In Table 6, we report on several indicators related to the coverage of health service interventions among KPs in the consistent locations. A large majority of respondents reported having ever been tested for HIV. Comparing the second to the first round, there was a significant increase in reported HIV testing in the last three months among FSWs (81% vs. 72%, p<0.001). There were also significant increases among FSWs (69% vs. 53%, p<0.001) and MSM (69% vs. 64%, p<0.05) who reported receiving services from a project clinic or a drop-in centre (DIC).

**Table 6:** Reported HIV testing and utilization of health services among polling booth survey participants, 2014 and 2015, consistent locations.

Behaviour	FSWs			MSM			PWID		
	Consistent locations			Consistent locations			Consistent locations		
	2014 (%)	2015 (%)	p-value	2014 (%)	2015 (%)	p-value	2014 (%)	2015 (%)	p-value
**HIV test**									
Ever tested for HIV	94	96	p<0.01	92	94	p<0.05	94	93	p = 0.8
Tested for HIV in the past three months	72	81	p<0.001	-	77	-	71	75	p = 0.09
**Visit to Clinic**									
Visited or received services from the DIC or project clinic in the last three months	53	69	p<0.001	64	69	p<0.05	71	74	p = 0.3

New questions were added in the second round of PBS regarding HIV positivity and linkage to care outcomes. 24% of FSWs, 22% of MSM and 19% of PWID reported knowing that they were HIV positive in the consistent locations. Enrolment in ART programmes was reported by 80% of the FSWs, 70% of the MSM and 73% of the PWID who reported being HIV positive.

### Structural outcomes

As shown in Table 7, comparing the second to the first round, there were decreases in reported forced sex in the past six months among FSWs (18% vs. 22%, p<0.001) and MSM (13% vs. 17%, p <0.05). Reported police-related violence among FSWs increased significantly (49% vs. 44%, p<0.001)). However among PWID, there was a significant reduction in reported police arrests (45% vs. 57%, p<0.001).

**Table 7:** Reported experience of violence among polling booth survey participants.

Behaviours	Key populations								
	FSWs			MSM			PWID		
	Consistent locations			Consistent locations			Consistent locations		
	2014 (%)	2015 (%)	p-value	2014 (%)	2015 (%)	p-value	2014 (%)	2015 (%)	p-value
Forced to have intercourse in the past six months	22	18	p<0.001	17	13	p<0.05	8	7	p = 0.7
Arrested or beaten by police in the past six months	44	49	p<0.001	24	26	p = 0.25	57	45	p<0.001

## Discussion

Our results provide a barometer of the progress made by Kenya’s HIV combination prevention programming among key populations. It is important for programmes taking a location-population driven approach to understand for whom interventions should be targeted, and the facets of their HIV risk and vulnerability [17]. Our findings confirm that key populations in Kenya face diverse as well as overlapping dimensions of risk and vulnerability for HIV infection [1]. All female sex workers who participated in this survey sold sex in the last three months, while a much lower proportion of MSM respondents sold sex to men in the last one month, similar to the results in 2014. Almost one-sixth of female injecting drug user respondents also sold sex to paying clients in the last three months. A quarter of the male PWID respondents bought sex and were clients of sex workers, thus compounding their HIV risk beyond sharing of needles. While anal sex is high among MSM, as expected, 12% of FSWs and 6% of PWID also reported engaging in anal sex in the last month. While 91% of PWID injected heroin or other narcotic drugs in the last one month, 6% of FSWs and 10% of MSM respondents also reported injecting heroin or other narcotic drugs in their lifetime. These data indicate that key populations in Kenya have overlapping risks, and there is a need to address multiple risk behaviours among sub-populations. We now discuss the areas where progress was most and least apparent in the PBS outcomes between 2014 and 2015, and implications for better integrating the combination of behavioural, biomedical and structural interventions of the programme to sustainably achieve its targets.

The main areas of improvement were observed with respect to condom use and needle exchange, as well as HIV testing and treatment. First, there were consistent improvements in the availability as well as utilization of condoms with clients among both FSWs and MSM. Likewise, the practice of having sex under the influence of alcohol declined, as well as condom breakage, which others have shown to be interrelated [18–22]. The feasibility of increasing condom use among key populations, through the constant availability of free condoms as well as regular demonstrations by peer educators, has been demonstrated in other settings [23–26]. Evidence from Kenya and elsewhere supports that higher levels of exposure to peer educators are linked to higher levels of consistent condom use and improved HIV knowledge [27–32]. Although peer contact was not measured in 2014, results from the 2015 PBS show that peer contact was high in all three sub-populations. Hence, high peer contact coupled with increased availability of condoms, improved skills for using condoms, and reduced sex acts under the influence of alcohol, may have resulted in the observed increases in condom use at last sex with a paying client among FSWs and MSM. Among PWID, sharing of needles has declined and clean needle availability has increased. This may be due to expanded clean needle/syringe distribution at times and venues convenient for PWID, as shown in other settings [33].

In addition to behavioural interventions, the combination of HIV testing and antiretroviral therapy is an important strategy for HIV prevention and care. Our data show that ever testing for HIV and testing in the last three months have increased among KP programmes across the country. This finding is consistent with other successful programmes that have increased HIV counselling and testing among sex workers [28, 34, 35]. In Kenya, rapid Voluntary Counselling and Testing (VCT) provided through multiple channels, such as health facilities, drop-in centres and outreach clinics, has been associated with a large increase in uptake of HIV-testing and receipt of results [36]. Kenya is also hopeful that the launch of HIV self-testing guidelines and expansion of self-testing will make HIV testing even more accessible for key populations by providing more privacy and confidentiality, although challenges in providing subsequent linkages to counselling as well as to treatment will need to be addressed [37].

The PBS results in 2015 also showed that enrolment of HIV positive KP members in ART programmes was high, from 69–80% across all three KP populations. ART uptake among KP members has been low in other settings. Reports by UNAIDS indicate that only an average of 24% of HIV-positive people who inject drugs, 21% of HIV-positive sex workers and 14% of HIV-positive men who have sex with men were enrolled in ART in 2015. This was much lower than UNAIDS estimates of global coverage of ART among all HIV-infected persons, which was 46% in 2015 [38, 39]. The “test and treat” strategy that was adopted by Kenya recently to reduce the time taken to initiate ART among people living with HIV (PLHIV) should be leveraged by the key population programme to further improve effective and efficient linkage to ART for KP members [40]. Exposure to HIV prevention programmes, drop-in centres and health clinics has increased since 2014 for all KP populations. This is an encouraging trend, as clinic visits, peer educator contact, and supportive networks have been found to be strongly associated with the willingness of KP members to engage in HIV testing, care and treatment programmes, as well as in ART initiation and adherence [29, 31, 39, 40].

Despite the above improvements, condom use with non-paying partners among FSWs and MSM has remained low, drug overdoses among PWID has remained high, and violence by police towards FSWs has increased. The finding of low condom use with regular non-paying partners in this context is consistent with other studies, and is known to pose substantial health risks to key populations [41–43]. Behavioural interventions were shown to be ineffective in improving condom use with non-paying partners in a study from Mexico [31]. A study from Ethiopia has shown the mutual reinforcement of non-condom use in non-paying partnerships with higher alcohol use and client violence [34]. High levels of social and economic dependency on regular partners have also been found to be an underlying reason for inconsistent condom use with regular, non-paying partners [44]. Qualitative studies in Nepal and south India have explored the experiences of FSWs in which their intimate partners or husbands would threaten to leave them or enact violence if they asked to use condoms. Another reason for non-condom use was that both intimate partners felt it was unnecessary to use condoms in trusting or “exclusive” relationships, in contrast to sex work with paying clients. These issues, combined with women’s dependence on their intimate partners’ financial support and social status, from which they and their children often benefited, serve to reduce condom use in the context of regular partnerships [45, 46].

A high observed prevalence of drug overdose among PWID also deserves attention, not only in its own right, but because it may signal the existence of wider barriers. Drug overdose may be higher when people are injecting in secluded environments, due to fear of criminalization and stigmatization [47, 48]. The high level of overdose suggests that social and structural issues must be addressed through promoting community-level change around social norms, and enabling legal environments that support, rather than counteract, public health behaviour change strategies [47, 48]. A strong effort by the public health system and clinics serving PWID must be made to continually train staff in overdose management, and the required number of new needles must be routinely quantified. Strong evidence of the benefits of needle-syringe exchange for preventing HIV transmission supports the continued investment in these interventions, combined with interventions to establish the supportive legal and policy environment required to ensure they are effective [47, 49].

The finding that there was a significant reduction in police violence against PWID, and in physical and sexual violence against MSM and FSWs, is encouraging. However, violence perpetrated by state actors, including police and *askaris* (administrative police in the counties), increased significantly for FSWs between the two PBS rounds. It is widely recognized that structural interventions to reduce high levels of police violence and incarceration are required to sustain gains from behavioural or biomedical preventive interventions for HIV [50]. Abuses of human rights perpetrated by state and non-state actors directly or indirectly increase KP vulnerability to HIV [51]. Yet programmes with key populations in several countries have been successful in reducing violence against them through effective and consistent structural interventions [52, 53]. In Kenya, the national programme and its partners need to continue to prioritize violence prevention and response activities at-scale.

As discussed in our earlier paper [5], the PBS methodology has several limitations. Due to its anonymous nature, responses cannot be linked with participants’ socio-demographic characteristics. However, we have tried to address this issue by stratifying participants into locations and typologies. Furthermore, PBS only allows for binary responses, thus limiting the depth of information that can be analysed. Some of the socio-demographic characteristics of the sample of FSWs differed slightly between the two rounds, which could not be adjusted for due to the nature of the PBS design. However, a systematic random sampling approach within each primary sampling unit was used in an attempt to ensure representativeness. Sampling bias could have resulted if peer educators, as members of the community, recruited only those they knew had used the programme services. However, the data suggests that such bias was not great because a sizeable proportion of this sample reported not using the services in the last quarter. We were also unable to use our data to compare both the sero-status and reported testing of individuals, to ensure that those tested were not already HIV positive. This may have led to underestimation of the number of newly-diagnosed people undergoing testing in the population. Finally, we do not have comparison data on the indicators added during the second PBS round; we intend to address this issue in the next PBS round. In spite of its limitations, PBS remains a rapid, accessible, and inexpensive methodology that can yield reliable data. As such, PBS approaches are suitable for monitoring programme outcomes on a regular basis, and can guide the continuous refinement of existing HIV prevention programming as contexts and priorities change over time.

## Conclusion

The focus on HIV prevention among key populations has never been as urgent as today to reach global HIV/AIDS targets [19]. In Kenya, the implementation of the KASF 2014/15–2018/19 requires robust monitoring and evaluation to track progress towards reduced HIV risk among key populations for which interventions are targeted. The data from the second round of the annual PBS survey show areas in which the key population interventions are making progress. The results suggest that there have been improvements in condom use with clients among FSWs and MSM, and new needle and syringe use among PWID. There has also been increased HIV testing, reduced physical and sexual violence towards FSWs and MSM, and police violence towards PWID. However, some areas appear to be more resistant to change, including condom use with non-paying partners of FSWs and MSM, drug overdose among PWID, ART initiation among HIV positive individuals, and the ongoing experience of violence from law enforcement officials by FSWs. Addressing the latter issues will require concerted efforts to create policy, legal and social environments that are more favourable for HIV prevention. These results underline the importance of continued investment in the combination prevention approach taken by Kenya’s KP programme to maintain momentum towards achieving its HIV prevention 2020 roadmap [19].
